# Circular Array of Magnetic Sensors for Current Measurement: Analysis for Error Caused by Position of Conductor

**DOI:** 10.3390/s18020578

**Published:** 2018-02-14

**Authors:** Hao Yu, Zheng Qian, Huayi Liu, Jiaqi Qu

**Affiliations:** School of Instrumentation Science and Opto-Electronics Engineering, Beihang University, Beijing 100191, China; yuhaoby@buaa.edu.cn (H.Y.); liuhy_one@163.com (H.L.); qujiaqi@buaa.edu.cn (J.Q.)

**Keywords:** circular array, current measurement, un-center, un-perpendicular, tunnel magnetoresistance sensors

## Abstract

This paper analyzes the measurement error, caused by the position of the current-carrying conductor, of a circular array of magnetic sensors for current measurement. The circular array of magnetic sensors is an effective approach for AC or DC non-contact measurement, as it is low-cost, light-weight, has a large linear range, wide bandwidth, and low noise. Especially, it has been claimed that such structure has excellent reduction ability for errors caused by the position of the current-carrying conductor, crosstalk current interference, shape of the conduction cross-section, and the Earth’s magnetic field. However, the positions of the current-carrying conductor—including un-centeredness and un-perpendicularity—have not been analyzed in detail until now. In this paper, for the purpose of having minimum measurement error, a theoretical analysis has been proposed based on vector inner and exterior product. In the presented mathematical model of relative error, the un-center offset distance, the un-perpendicular angle, the radius of the circle, and the number of magnetic sensors are expressed in one equation. The comparison of the relative error caused by the position of the current-carrying conductor between four and eight sensors is conducted. Tunnel magnetoresistance (TMR) sensors are used in the experimental prototype to verify the mathematical model. The analysis results can be the reference to design the details of the circular array of magnetic sensors for current measurement in practical situations.

## 1. Introduction

The non-contact current measurement technology are continuously developing. One hand, high performed linear magnetic sensor and signal conditioning IC for closed—loop magnetic current sensor have been lunched, e.g., DRV5055 and DRV401-EP form TI. On the other hand, new types of current sensors are been widely studied, e.g., Anisotropic magnetoresistance (AMR) sensors, Giant magnetoresistance (GMR) sensors [1], Tunnel magnetoresistance (TMR) sensors, magneto-optical sensors and superconduction current sensors [2]. In order to improve the measurement accuracy, the structure based on a circular array of magnetic sensors has being frequently studied during the past two decades [3,4,5,6,7,8,9,10,11,12]. Compared to other structures (e.g., open-loop or close-loop with magnetic cores, current transformers, etc. [13]), the circular array of magnetic sensors is considered to be an effective scheme to achieve low-cost, large linear range, wide frequency bandwidth [14,15,16], light weight, and high reliability. Especially, the circular array of magnetic sensors can be used for both DC and high-frequency AC measurement, compared to Rogowski coil [17], which can only be used for high-frequency and low frequency (50/60) current measurement. However, the measurement accuracy of this structure mainly suffers from the error caused by position of the current-carrying conductor and the crosstalk current interference. Much research has focused on the relationship between the error reduction ability and the parameters of the circular array of magnetic sensors.

The installation position offset of the current-carrying conductor influences the accuracy of the magnetic sensor circular array, including wire un-centeredness and un-perpendicularity. In [6], the relative measurement error dependence on the displacement of the conductor from the center of the circle has been discussed, and the displacement angles between the first sensor to the *x*-axis ($\alpha_{0}$) was also considered in the theoretical analysis. Different from other works, AMR sensors were applied. Recently, Pavel Ripka et al. also presented a method of calibration and error correction for the circular array of AMR sensors. After the calibration phase, the off-center error was reduced from 0.4 to 0.06% [12].

The effect of external magnetic fields is considered to be one of the important factors that limits the measurement accuracy, which has been generally discussed. An algorithm based on discrete Fourier transform (DFT) to improve the crosstalk reduction has been proposed, which can be realized on a Digital Signal Processor (DSP) or other microcontroller [4]. In their work, a circular array of eight Hall sensors was used to verify the efficiency of the crosstalk error reduction algorithm. In another literature, the external magnetic fields was reduced using a spatial circular harmonic expansion of the magnetic scalar potential [7]. Then, Roland Weiss et al. [8] improved the modeling and the experimental setup of the above method, and verified it with six fluxgate sensors. The effect of different displacement angles between the first sensor to the *x*-axis ($\alpha_{0}$) was discussed. They also analyzed the effect of the geometry of different flat conductors in another work [9], and achieved current errors of less than 1.5%. The latest research work of Pavel Ripka et al. [11] investigated the effect of external current on circular array of magnetic sensors and analysed the influence of real conductor size and uneven density of AC currents. If the geometry of the external conductor is known, the compensation of the cross-sensitivity can be calculated.

However, although the measurement caused by un-centering has been discussed, no researcher has addressed the problem of the conductor un-perpendicularity until now. Unfortunately, the conductor un-perpendicularity is not an ignorable factor, especially in the situation where the conductor is flexible, and usually it may combined with the un-center offset. Based on above issues, this paper used the inner and exterior products in vector space and analyzed the error caused by the current-carrying conductor position (which includes un-centeredness and un-perpendicularity). The experiment based on high-performance commercial TMR sensors has been conducted to verify the theoretical model. The combination effect is also discussed in our work. Finally, the allowable range of un-centeredness offset and un-perpendicularity angle can be given by the mathematic model.

## 2. Mathematical Background

**Figure 1:**
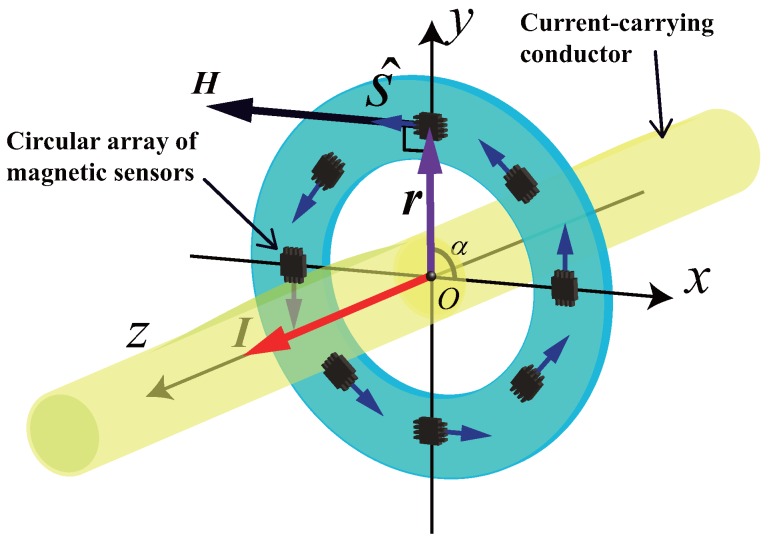
Basic theory of the circular array of eight magnetic sensors.

The structure of the circular array of magnetic sensors is shown in Figure 1—eight or some other number of magnetic sensors uniformly arranged in a circle with radius *r*. The sensors can be installed on a printed circuit board (PCB), and the sensitivity directions are always perpendicular to r—the vector from the center to the sensor sensitivity point. The current-carrying conductor—which is strictly straight and has a proximate infinite length—crosses from the center of the circle. In a three-dimensional Cartesian coordinate system, according to Biot–Savart law [18], the output signal of an individual magnetic sensor, *V*, can be expressed by

(1) $$\begin{array}{c} V=k_{s}\left(H\cdot \hat{s}\right)=k_{s}\frac{I\times r}{2\pi r^{2}}\cdot \hat{s}, \end{array}$$

where, $k_{s}$ is the sensitivity parameter of the magnetic sensors (assuming the sensors are linear and totally in accordance), H is the vector of the magnetic field generated by the current-carrying conductor, I is the current vector in the conductor, $\hat{s}$ is the unit vector ($|\hat{s}|=1$) of the sensitivity direction of the magnetic sensor. In (1), according to the definition of an exterior product, I×r means the result is a vector which is both perpendicular to I and r, and follows the right-hand rule. At the same time, according to the definition of an inner product, $H\cdot \hat{s}$ means the result is the projection value of the vector H on the direction $\hat{s}$; it represents the fact that the output of the magnetic sensor only relates with the magnetic field along the sensitivity direction.

In a circular array constructed by *N* magnetic sensors, the mean value of the sensor outputs can be expressed as

(2) $$\begin{array}{cc} V_{mean}=\frac{1}{N}\sum_{n=0}^{N-1}V^{\left(n\right)}= & \frac{k_{s}}{N}\sum_{n=0}^{N-1}\left(H^{\left(n\right)}\cdot {\hat{s}}^{\left(n\right)}\right)=\frac{k_{s}}{N}\sum_{n=0}^{N-1}\frac{I\times r^{\left(n\right)}}{2\pi {\left(r^{\left(n\right)}\right)}^{2}}\cdot {\hat{s}}^{\left(n\right)}, \end{array}$$

where the superscript (n) represents the *n*th sensor parameters; for instance, ${\hat{s}}^{\left(1\right)}$ is the sensitivity direction of the first magnetic sensor. $V_{mean}$ can be easily measured and calculated, $k_{s}$ can be a constant after calibration, and the measured I is the only unknown quantity in (2). If the current-carrying conductor is offset from the center or is not perpendicular to the circle plane, $r^{\left(n\right)}=\left|r^{\left(n\right)}\right|$ will have different values; otherwise, they will be equal ($r^{\left(n\right)}=r$) in ideal conditions. Therefore, the calculated current value, $I_{cal}$, of the under measured current can be calculated by

(3) $$I_{cal}=\frac{2\pi rV_{mean}}{k_{s}},$$

and the relative measurement error is

(4) $$\varepsilon =\frac{\left|I_{cal}-I\right|}{I}\times 100\%.$$

## 3. The Error Analysis

### 3.1. The Mathematical Model of the Errors from the Un-Centeredness and Un-Perpendicularity

**Figure 2:**
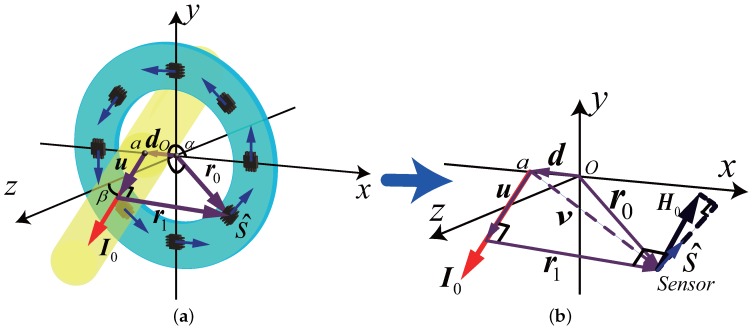
The error caused by the current-carrying conductor position: (**a**) Schematic of un-centeredness and un-perpendicularity; (**b**) The relationship of the key vectors

Un-centeredness and un-perpendicularity of the current-carrying conductor may coexist in practical situation. The modelling method introduced in the mathematical background is useful for analysis of the measurement errors caused by these factors. Figure 2 shows the magnetic field generated by the under-measured current $I_{0}$, which crosses the offset point *a* from the center, and is un-perpendicular to the circle plane. The parameter *d* presents the offset distance from the circle center, and β presents the un-perpendicularity angle from the *z*-axis, where $d<r_{0}$ and 0<β<π/2. The definitions and values of the vectors in Figure 2 are listed in Table 1. Note that the superscript (n) is not used for the moment.

Additionally, the norm of u is the projection of v on $I_{0}$, according to the definition of vector inner product, u can be calculated by

(5) $$u=\left|u\right|{\hat{I}}_{0}=\left(v\cdot {\hat{I}}_{0}\right){\hat{I}}_{0}=\left[\begin{array}{c} \sin^{2}\beta \left(r_{0}\cos\alpha -d\right)\\ 0\\ \sin\beta \cos\beta (r_{0}\cos\alpha -d) \end{array}\right].$$

From the vectors relationship schematic diagram in Figure 2, the vector from $I_{0}$ to the sensor can be calculated by

(6) $$\begin{array}{c} r_{1}=r_{0}-d-u=\left[\begin{array}{c} \cos^{2}\beta \left(r_{0}\cos\alpha -d\right)\\ r_{0}\sin\alpha \\ \sin\beta \cos\beta (d-r_{0}\cos\alpha ) \end{array}\right]. \end{array}$$

According to (2), the mean value of the output signal of the sensors can be straightforwardly written as

(7) $$\begin{array}{cc} V_{mean} & =\frac{k_{s}I_{cal}}{2\pi r_{0}}=\frac{1}{N}\sum_{n=0}^{N-1}V^{\left(n\right)}=\frac{k_{s}}{N}\sum_{n=0}^{N-1}\left({H_{0}}^{\left(n\right)}\cdot {\hat{s}}^{\left(n\right)}\right)=\frac{k_{s}}{N}\sum_{n=0}^{N-1}\frac{I_{0}\times r_{1}^{\left(n\right)}}{2\pi {\left(r_{1}^{\left(n\right)}\right)}^{2}}\cdot {\hat{s}}^{\left(n\right)}\\ & =I_{0}\frac{k_{s}}{2\pi N}\sum_{n=0}^{N-1}\frac{\cos\beta (r_{0}-dcos\alpha^{\left(n\right)})}{\cos^{2}\beta {\left(r_{0}\cos\alpha^{\left(n\right)}-d\right)}^{2}+r_{0}^{2}sin^{2}\alpha^{\left(n\right)}}, \end{array}$$

where $\alpha^{\left(n\right)}$ is the angle between the *n*th sensor position vector ${r_{0}}^{\left(n\right)}$ and the +x-axis, which is expressed by

(8) $$\alpha^{\left(n\right)}=\frac{2\pi n}{N}+\alpha_{0}\;\;\;\;\left(n=0,1,...,N-1\right),$$

where $\alpha_{0}$ is the offset angle between the magnetic sensor array and the +x-axis, which is a non-negligible parameter affecting the measurement error (refer to [6,9]). We will analyze the effect of $\alpha_{0}$ later.

The calculated current $I_{cal}$ in (7) is the calculated current by the circular array of magnetic sensors, which can be written as

(9) $$\begin{array}{c} I_{cal}=I_{0}\underset{\Delta }{\underset{︸}{\frac{r_{0}}{N}\sum_{n=0}^{N-1}\frac{\cos\beta (r_{0}-dcos\alpha^{\left(n\right)})}{\cos^{2}\beta {\left(r_{0}\cos\alpha^{\left(n\right)}-d\right)}^{2}+r_{0}^{2}sin^{2}\alpha^{\left(n\right)}}}}=I_{0}\Delta , \end{array}$$

where Δ is the key part that leading the measurement error, and it can be proved that

(10) $$\begin{array}{c} \lim_{N\to +\infty }\Delta =1. \end{array}$$

Therefore, Equation (10) leads to $I_{cal}$ being approximately equal to the actual under-measured current $I_{0}$, which theoretically proves that the sum of the output of the circular array is an approximation of Ampere’s circulation when N→+∞. According to the method proposed by Weiss et al. [8], the relative error $\varepsilon /I_{0}$ is used to present the effect caused by *d* and β, which actually is the Δ in our Equation (9). Therefore, the relative measurement error caused by *d* and β is

(11) $$\begin{array}{cc} \varepsilon_{d\beta } & =\frac{I_{cal}-I_{0}}{I_{0}}\times 100\%=\Delta -1=\frac{r_{0}}{N}\sum_{n=0}^{N-1}\frac{\cos\beta (r_{0}-dcos\alpha^{\left(n\right)})}{\cos^{2}\beta {\left(r_{0}\cos\alpha^{\left(n\right)}-d\right)}^{2}+r_{0}^{2}sin^{2}\alpha^{\left(n\right)}}-1. \end{array}$$

Finally, for convenience, the situation of un-centeredness and un-perpendicularity are discussed, respectively. For β=0, the relative measurement error caused by *d* is

(12) $$\begin{array}{c} \varepsilon_{d}=\frac{r_{0}}{N}\sum_{n=0}^{N-1}\frac{r_{0}-d\cos\alpha^{\left(n\right)}}{r_{0}^{2}+d^{2}-2r_{0}d\cos\alpha^{\left(n\right)}}-1, \end{array}$$

and for d=0, the relative measurement error caused by β is

(13) $$\begin{array}{c} \varepsilon_{\beta }=\frac{1}{N}\sum_{n=0}^{N-1}\frac{\cos\beta }{\left(1-cos^{2}\alpha^{\left(n\right)}\sin^{2}\beta \right)}-1. \end{array}$$

Note that $r_{0}$ does not exist in $\varepsilon_{\beta }$, meaning that $\varepsilon_{\beta }$ has no relationship with $r_{0}$.

### 3.2. Analysis of the Effect of Displacement Angles of the Sensor Array

**Figure 3:**
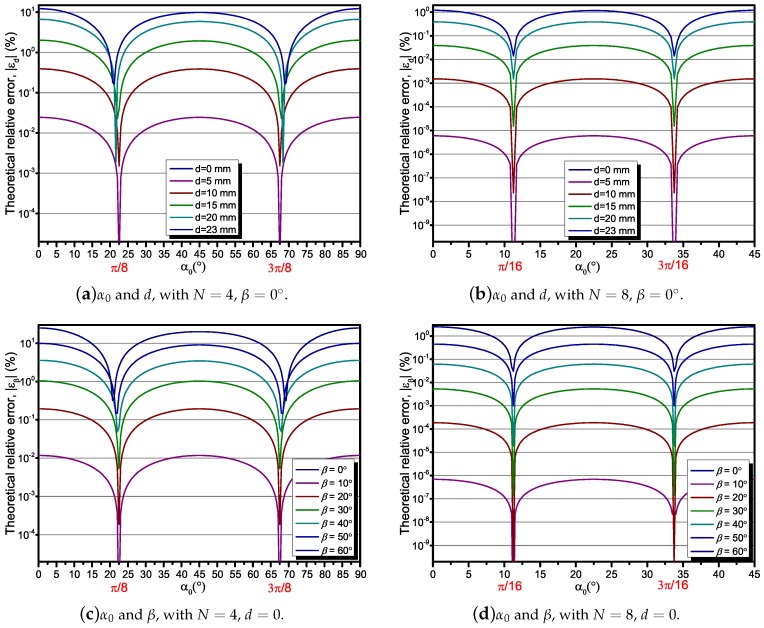
The theoretical relative error analysis depending on $\alpha_{0}$, β, and *d*, with $r_{0}=40$ mm.

To analyze the effect of the displacement angles of the sensor array $\alpha_{0}$ on $\varepsilon_{d}$ and $\varepsilon_{\beta }$, we calculated the $\varepsilon_{d\beta }$ with the sensor number N=4 and N=8. In our case, $r_{0}=40$ mm. In Figure 3a,b, it can be seen that for *d* ranges from 0 mm to 23 mm, the relative error $\varepsilon_{d}$ reaches a max point when $\alpha_{0}=2\pi n/N$, and a min point approximately when $\alpha_{0}=\pi \left(2n+1\right)/2N$. Note that for d=23 mm, the maximum $\varepsilon_{d}$ reduces from 12.27% to 1.21%, while *N* increases from 4 to 8. The same above results can also be found in Figure 3c,d, while d=0, β ranges from 0° to 60°.

Based on the above analysis, it can be concluded that the change of $\alpha_{0}$ can reduce the relative error effectively. The relative error may be particularly reduced to a small level that can be ignored. However, unfortunately for practical situations, the position of the conductor is usually uncertain, and so is the $\alpha_{0}$. Therefore, we keep the $\alpha_{0}=0^{\circ }$ in the rest of our analysis in order to study the maximum effect on measurement accuracy of the position error of the current-carrying conductor.

### 3.3. Analysis of the Effect of Un-Centeredness and Un-Perpendicularity

**Figure 4:**
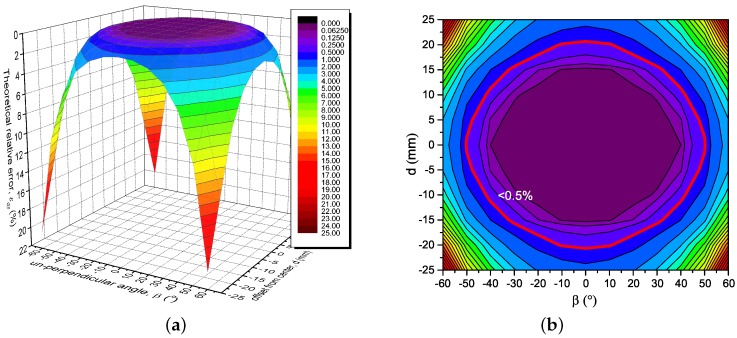
The theoretical relative error depending on *d* and β, N=8, $\alpha_{0}=0^{\circ }$. (**a**) 3-D view; (**b**) The contour line, red circle is the contour line of 0.5%.

The un-centeredness and un-perpendicularity of the current-carrying conductor usually coexist in practical situations. Especially, the effect of the conductor position error will become increasingly difficult to ignore in the situation where the conductor is a soft wire, which may not be strictly straight and fixed in a certain position. For that reason, the combination of the relative error, $\varepsilon_{d\beta }$, caused by un-centeredness and un-perpendicularity become more necessary to consider together. In Figure 4, $\varepsilon_{d\beta }$ is calculated by Equation (11) with N=8, $r_{0}=40$ mm; it can be seen that the relative errors are retained within 0.5% of the major region of *d* and β. With the approximate region of −10 mm<d<10 mm and $-30^{\circ }<\beta <30^{\circ }$, the relative errors stay within 0.077%, and increase rapidly as the absolute value of *d* and β both increase. For the extreme situation, $\varepsilon_{d\beta }$ increases to 20.25% with $\beta =\pm 60^{\circ }$, d=±23 mm, and N=8. Table 2 lists the values of $\varepsilon_{d\beta }$ with *N* ranges from 2 to 16 with different *d* and β. From the table, extreme $\varepsilon_{d\beta }$ can be reduced to 2.896% by increasing *N* to 16, and becomes an ignorable level within the region of −10 mm<d<10 mm and $-30^{\circ }<\beta <30^{\circ }$.

## 4. Experimental Procedure

**Figure 5:**
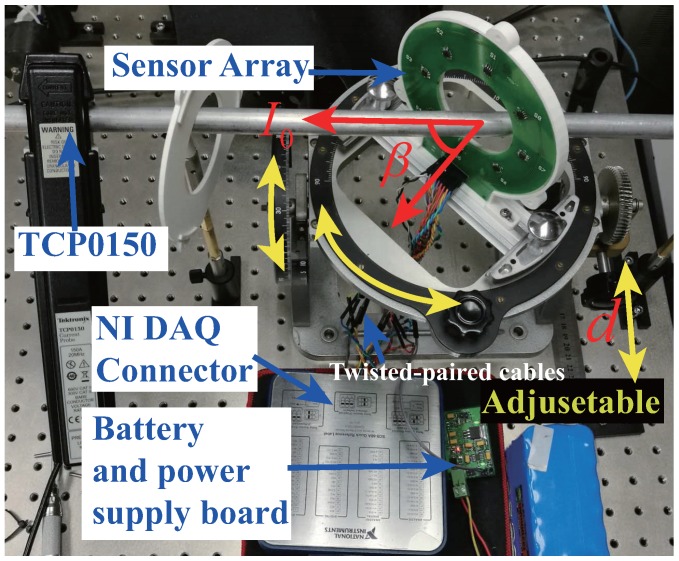
Experimental setup with adjustable platform.

The experimental setup is shown in Figure 5. Four or eight commercial TMR sensors (TMR2103) were placed on an annular PCB. The circle radius of the sensor array was 40 mm. The TMR2103 was manufactured by MultiDimension Technology (MDT), with the linear measurement range of ±30 Oe [19] and high sensitivity of 6 mV/V/Oe (1 Oe = 1 Gauss in air = 0.1 millitesla = 79.8 A/m). TMR2103 includes four magnetic tunnel junction (MTJ) elements constructing a Huygens bridge. In comparison with Hall effect sensors, AMR sensors, GMR sensors, and other magnetic sensors, the TMR sensor has higher sensitivity, better temperature stability, lower power consumption, and better linearity [20,21]. Especially, the TMR sensor has a higher frequency range [22], which is an advantage for higher-frequency AC or transient current measurement.

The output of TMR2103 was differential and connected to a PCI DAQ system of National Instruments (NI PXIe-6366) through PCB wires and twisted-paired cables. Differential signals might effectively reduced the affect from the cables and the spatial distribution interference. The signals were read and processed via LabVIEW software. The current source (AHY-15-10-200) can provide a stable maximum 200 A current with a frequency range of 40 to 600 Hz. The current in the current-carrying conductor was also measured by a TCP0150 current probe with an accuracy of 0.01% and a frequency range of DC to 2 MHz, which could be treated as a reference current sensor in our case. With the purpose of reducing the power noise, the power of the sensor array was supplied by a battery through a linear DC stabilizer voltage supply board. The system diagram of the experimental setup was similar to the proposed work by Renzo Bazzocchi et al. [4]. Furthermore, we improved that by an adjustable shelf so that the *d* and β could be adjusted conveniently and accurately. The yellow arrows in Figure 5 indicate that the equipment is adjustable along the pointed directions. Most components in the setup, including the adjustable shelf and the optical platform, are made of non-ferromagnetic materials in order to ensure that the stray magnetic field interference was minimized.

Firstly, the calibration was conducted with five separate measurement cycles with the current from 10 A to 140 A and back at a frequency of 400 Hz. The output signals of all the sensors in the array and the measurement results of the TCP0150 were detected. The root-mean-square (RMS) of each signal was calculated. Then, the sensors’ outputs and the reference result from TCP0150 were fit by the linear least-squares method in LabVIEW and Matlab. After that, the differences in the sensitivity of all TMR sensors could be minimized. In this procedure, the current-carrying conductors were strictly crossing the center of the circle and perpendicular to the sensor array’s plane.

Following the calibration, $\beta =0^{\circ }$ was maintained, for the *d* changing from −24 mm to 24 mm with steps of about 5 mm, and multiple RMS measurements of the output signals of the TMR sensor array were conducted. After that, the same procedure was conducted for β changing from $-60^{\circ }$ to $60^{\circ }$, keeping d=0. Note that we kept $\alpha_{0}=0$ in all procedures, because only the maximum relative error was considered in our analysis.

## 5. Results

### 5.1. Calibration Results

**Figure 6:**
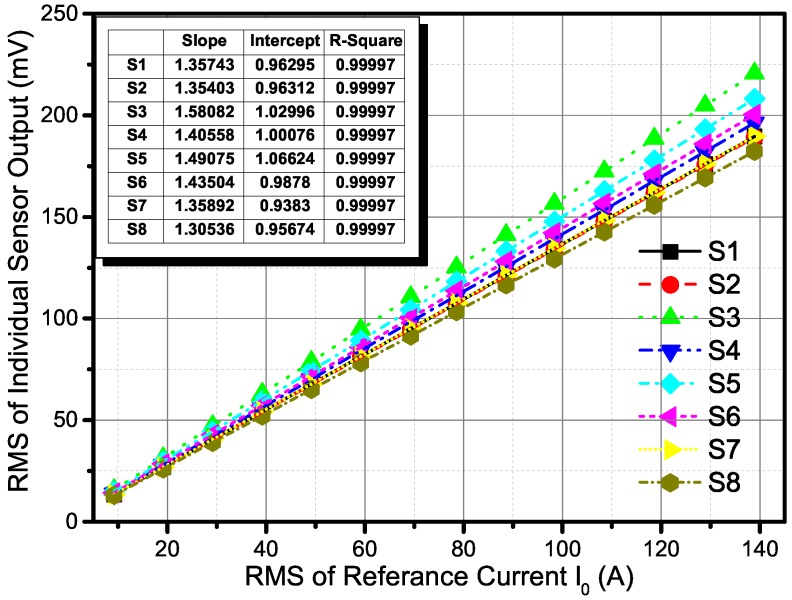
Calibration result for individual outputs of sensors. The results of the linear fit for individual sensors are listed in the table.

The calibration for individual sensors was conducted as presented in the experimental procedure section. In Figure 6, the curves were fit by the linear least-squares method and the results are listed. It can be seen that the differences between the eight TMR sensors are obvious, which also exist in other kinds of magnetic sensors (e.g., GMR sensors, AMR sensors, or Hall sensors). After fitting the individual sensor output characteristics, the parameters were used to calculate every sensor measurement result, so that the differences were effectively minimized. This validates the assumption in the mathematical model that the sensitivities of sensors $k_{s}$ were equal.

### 5.2. The Result of Un-Centeredness and Un-Perpendicularity

**Figure 7:**
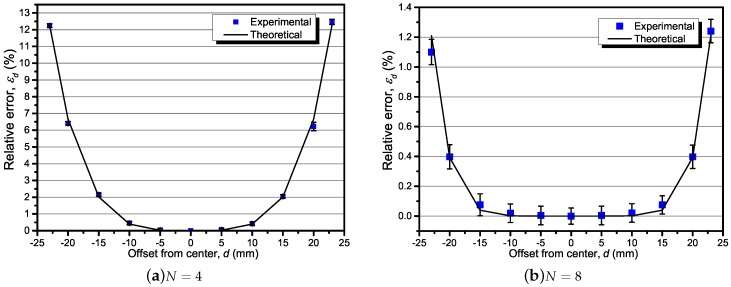
The un-center effect with various offset distances *d* from −23 mm to 23 mm, with $\beta =0^{\circ }$, $\alpha_{0}=0^{\circ }$, and test current *f*= 400 Hz @ 50 A.

According to the procedure proposed in the experimental procedure section, the theoretical and experimental results of the relative error caused by un-centeredness and un-perpendicularity were obtained. For *d* changing from −24 mm to 24 mm, the result of $\varepsilon_{d}$ is shown in Figure 7a with N=4, and in Figure 7b with N=8. It can be seen that $\varepsilon_{d}$ achieved the maximum value for d=±23 mm and retained a small value in the range of −10 mm to 10 mm. The theoretical results were verified by the experimental results with four and eight TMR sensors. With −10 mm<d<10 mm, the relative error $\varepsilon_{d}$ reduced below 0.2% (for example) in the N=8 cases, much less than that in the N=4 cases. From another perspective, for the purpose of keeping relative error below 0.2% (for example), the allowable range of *d* would be expanded from ±5 mm to ±10 mm while increasing the number of sensors from 4 to 8.

**Figure 8:**
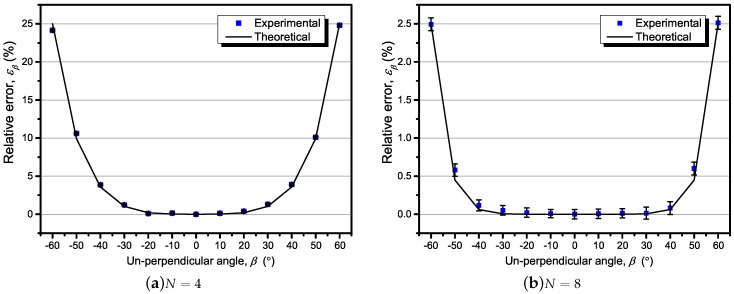
The un-perpendicular effect with the offset angle β varying from −60° to 60°, with d=0, $\alpha_{0}=0^{\circ }$, and test current *f*= 400 Hz @ 50 A.

The relative error caused by un-perpendicularity was measured and calculated, as shown in Figure 8a,b. The proposed theory was also well verified by the experimental results. For different β from −30° to 30°, the relative error caused by β stay within 0.2% with eight TMR sensors. It is illustrated that increasing the number of sensors led to a more accurate and reliable current measurement by the sensor array.

## 6. Discussion

However, there were still errors between the experimental and theoretical results, which might have been caused by the position error of the experimental setup—especially the adjustable shelf. Another reason is the fluctuation of the separation lengths between the TMR sensors, even the sensors were installed in the PCB by SMT component placement system. The experimental setup can be improved by using a high-precision electromotional translation stage which has multiple degrees of freedom. Other source of error may have been residual calibration error in individual TMR sensors, and the sensitivity axis error of the TMR sensors may also have contributed error (i.e., may not have been strictly perpendicular to $r_{0}$). Besides these, the fluctuation of the current source may also cause the different between the calculated and measured relative error.

Despite these errors, the experimental and theoretical results in this paper can be a reference for the design of circular arrays of TMR sensors for current measurement in practical cases. From Equation (11), the effect of the un-center offset *d*, the un-perpendicular angle β, the number of TMR sensors *N*, and the radius of the circle $r_{0}$ are obvious. At the same time, the linear range of the TMR sensors and the maximum under-measured current limit the radius of the circle by Equation (1). With the purpose of reducing the relative error $\varepsilon_{d\beta }$, the un-center offset *d* and un-perpendicular angle β can be limited by mechanical structure design. For instance, the gap between the current-carrying conductor and the shell of the sensors array can be minimized to limit the offset *d*. It is also possible to limit the un-perpendicular angle β effectively by increasing the thickness of the shell of the sensor array along the direction of the *z*-axis.

Furthermore, the relative error $\varepsilon_{d\beta }$ can be reduced below the usual level that can be neglected by increasing the number of magnetic sensors to 16 or more by the calculated result of Equation (11). It is necessary to have a tradeoff between the accuracy and the cost in an actual case. Although the analysis has been presented in the case of several conditions, there are also many factors that may cause measurement error which have not been discussed in this paper. For instance, the crosstalk current interference, the Earth’s magnetic interference, etc. For individual TMR sensors, their hysteresis [23,24,25,26], nonlinearity, bandwidth, temperature property, etc. cannot be neglected. In the application of a circular array of TMR sensors, the signal process circuit must be designed well to calibrate the individual sensors and output the sum of all the sensors. Because the approach proposed in this paper has the ability to measure DC to high-frequency current, further work will focus on the AC frequency response test and the extension of the frequency bandwidth of the circular array of magnetic sensors.

## 7. Conclusions

We analyzed the relative measurement error of a circular array of magnetic sensors caused by position error of the current-carrying conductor in this paper. For the purpose of achieving minimum measurement error, the theoretical results were proposed and verified by an experimental setup. The effects of un-center offset, un-perpendicular angle, number of magnetic sensors, and the radius of the circle on relative error are expressed in one equation. The allowable range of un-center offset and un-perpendicular angle are given to ensure the relative error is retained within an acceptable level. The relative measurement error can be reduced by limiting the displacement of the conductor. In the case of 8 TMR sensors, the relative measurement error can be retain within 0.2% with the un-center offset of ±10 mm and the un-perpendicular angle of $\pm 30^{\circ }$. For the un-center offset and un-perpendicular angle can not be measured easily in practice, the further work may be the estimation of the the un-center offset and un-perpendicular angle (and other parameters such as crosstalk current, etc.) using the output signals of the magnetic sensors, base on the relative measurement error model. 

## Figures and Tables

**Table 1:** Parameters and vectors in Figure 2.

Vector	Norm	Value	Definition
$I_{0}$	$I_{0}$	$I_{0}{\left(\sin\beta ,0,\cos\beta \right)}^{T}$	Under-measured current
d	*d*	${\left(d,0,0\right)}^{T}$	Offset vector from the center
$r_{0}$	$r_{0}$	$r_{0}{\left(\cos\alpha ,\sin\alpha ,0\right)}^{T}$ *	Position vector from *O* to sensor
v	*v*	$r_{0}-d$	Position vector from point *a* to sensor
$r_{1}$	$r_{1}$	v−u	Position vector from $I_{0}$ to sensor
u	*u*	see (5)	Position vector from *O* to $r_{1}$
$\hat{s}$ **	1	${\left(-\sin\alpha ,\cos\alpha ,0\right)}^{T}$	Sensitivity direction of sensor

* α is the angle between *x*-axis and $r_{0}$. ** $\hat{s}$ is perpendicular to $r_{0}$.

**Table 2:** Theoretical $\varepsilon_{d\beta }$ with *N* ranges from 2 to 16, $r_{0}=40$ mm.

*N*	$\varepsilon_{d\beta }$	$\varepsilon_{d\beta }$ (%) with
	$\beta =\pm 60^{\circ }$, d=±23 mm *	$\beta =\pm 30^{\circ }$, d=±10 mm
2	198.8	23.17
4	72.48	2.9464
6	36.05	0.4665
8	20.25	0.07696
10	12.04	0.01279
12	7.382	0.002129
14	4.601	0.0003544
16	2.896	0.000005899

* The extreme situation in our case.

## Abbreviations

The following abbreviations are used in this manuscript:

AMR	Anisotropic Magnetoresistance
GMR	Giant Magnetoresistance
TMR	Tunnel Magnetoresistance
MTJ	Magnetic Tunnel Junction
AC	Alternation current
DC	Direct current
RMS	Root mean square
